# Crucial Roles in Drosophila Development for Little-Known Protein

**DOI:** 10.1371/journal.pbio.0030139

**Published:** 2005-03-15

In the intricate dance of animal development, several critical steps are choreographed by the Notch receptor and its ligands, including Delta (Dl) and Serrate (Ser). When these ligands bind, an intracellular segment of the receptor is cleaved off and becomes a transcriptional regulator within the nucleus. While Dl and Ser both occur in Drosophila, and appear to have similar interactions with Notch, less is known about exactly how they trigger Notch activity. In particular, few details are known about the activators of Dl and Ser, which begin the cascade ultimately leading to Notch signaling. In this issue, François Schweisguth and colleagues show that two activators, Drosophila mind bomb (D-mib) and Neuralized (Neur), while sharing a similar molecular activity, have distinct roles in Notch-related Drosophila development, and elucidate several important activities of the heretofore mysterious D-mib.

D-mib and Neur are ubiquitin ligases, enzymes that attach the small protein ubiquitin onto a target protein. While ubiquitination was first appreciated as a tag for protein degradation, more recently, it has been recognized to be a signal for endocytosis, a process that brings substances outside a cell into the cell. Endocytosis, in turn, has recently been found to be a key step in Dl activation of Notch; its importance to Ser signaling has not been previously identified. In Drosophila, Neur targets Dl, and Dl is endocytosed. In zebrafish, Dl is a target for a separate ubiquitin ligase, Mind bomb. But the function of the Drosophila homolog, D-mib, has not been elucidated. Thus, the essential questions in this study were, what role does D-mib play in Drosophila, and how is Ser signaling regulated?

Schweisguth and colleagues studied development in a D-mib mutant. The pattern of phenotypic changes seen was in keeping with a loss of Dl signaling, indicating that D-mib interacts with Dl, just as Neur does. But D-mib also interacts with the other Notch ligand, Ser, as shown by the aberrant distribution of Ser in the absence of D-mib. Furthermore, endocytosis of Ser occurred normally in the presence of D-mib, and was strongly inhibited in its absence. Acting through Notch, Ser activates a downstream gene that codes for a protein called Cut, whose absence leads to a particular pattern of wing defects. This pattern was seen in D-mib mutants, and could not be rescued by overexpressing Ser, thus indicating that D-mib not only prompts endocytosis of Ser, but allows it to trigger Notch signaling. It's unclear whether D-mib ubiquitinates Ser, as this study did not specifically address that question.

While Neur and D-mib differ structurally, they share the same molecular function, ubiquitination of Notch ligands, and the authors show that, when ectopically expressed, each can at least partially compensate for the absence of the other. Nonetheless, they normally have distinct developmental functions, owing largely to the fact that they are expressed in different locations and at different times during development, and thus mutations in the two lead to different patterns of developmental aberrations.

